# The effects of different cooking modes on the ^137^Cs, ^40^K, and total K content in *Boletus edulis* (King Bolete) mushrooms

**DOI:** 10.1007/s11356-020-11147-7

**Published:** 2020-10-19

**Authors:** Martyna Saba, Jerzy Falandysz

**Affiliations:** 1 Environmental Chemistry & Ecotoxicology, University of Gdańsk, 80-308 Gdańsk, Poland; 2 Environmental and Computational Chemistry Group, School of Pharmaceutical Sciences, Zaragocilla Campus, University of Cartagena, Cartagena, 130015 Colombia

**Keywords:** Cs-137, Potassium-40, Potassium, Foods, Household processing, Mushrooms, Radioactive pollution

## Abstract

This study aimed to get an insight into the effects of household processing on the leaching behaviour of ^137^Cs and ^40^K from fresh, frozen and dried *Boletus edulis* (King Bolete) mushrooms. Three processes were investigated—blanching, blanching and pickling, and drying followed by grinding and soaking. The activity concentrations of ^137^Cs and ^40^K in the fresh unprocessed mushrooms were 270 Bq kg^−1^ dry biomass (27 Bq kg^−1^ whole weight) and 590 Bq kg^−1^ db (59 Bq kg^−1^ ww), respectively. Blanching of fresh mushrooms decreased ^137^Cs activity by 55%, and ^40^K activity by 34%, and blanching of deep-frozen mushrooms caused a reduction of 52% and 44% (db) (equivalent to whole weight reductions of 37% and 8.5%, and 67% and 22%, respectively). Blanching and pickling of fresh mushrooms decreased ^137^Cs activity by 83% and ^40^K activity by 87%, while blanching deep-frozen mushrooms resulted in decreases of 88% and 80% (db) (whole weight decreases of 77% and 81%, and by ~ 84% and 72%, respectively). This study confirms earlier reports that blanching of fresh or frozen mushrooms alone is not as efficient at removing ^137^Cs as blanching followed by pickling. The study also shows that the initial rate of fruiting body disintegration and pre-preparation (comparing fresh, deep-frozen, or dried and ground) can have an impact on the leaching rate of the water soluble fraction of metallic elements.

**Electronic supplementary material:**

The online version of this article (10.1007/s11356-020-11147-7) contains supplementary material, which is available to authorized users.

## Introduction

Macrofungi exhibit a remarkable aptitude and propensity to bioconcentrate a variety of major and trace mineral constituents, including radioactive elements, in their fruiting bodies (mushrooms) (Falandysz and Borovička 2013). Edible species that grow in the wild were considered as a potentially significant source of human dietary exposure to radiocaesium (^134/137^Cs) from the atmospheric fallout of radioactivity from nuclear weapon testing in the atmosphere and nuclear power plant accidents (Daillant et al. 2013; Kiefer et al. 1965; Rantavaara 2003). In particular, the incident at the Chernobyl nuclear power plant in 1986 led to ^137^Cs contamination of wild mushrooms in surrounding European regions (Betti et al. 2017; Daillant et al. 2013; Tucaković et al. 2018). Similarly, the incident at the Fukushima-Daichi nuclear power plant in 2011 caused ^137^Cs contamination of locally foraged mushrooms (Cui et al. 2020), without showing any noticeable effect on the wild species collected in continental Asia and Europe (Falandysz et al. 2018; Prand-Stritzko and Steinhauser 2018).

Mushrooms preparation techniques, e.g., blanching, boiling, parboiling, pickling, braising, stewing, grilling, frying, soaking (macerating), and also industrial conserving (pickling and canning), have an effect on the content of mineral constituents in processed products (Beresford et al. 2001; Drewnowska et al. 2017; Falandysz et al. 2020a, b; Pankavec et al. 2019; Skibniewska and Smoczyński 1999; Steinhauser and Steinhauser 2016; Stijve 1994). Daillant et al. noted that routine culinary practices using a variety of different treatments were only partly successful in decreasing ^137^Cs contents in potential mushroom meals (Daillant et al. 2013). Some practices such as stir-frying and braising cause an increase rather than diminution in ^137^Cs and ^40^K activity concentrations as well as concentration of mercury (Hg) compounds in the cooked products as the hot oil/processing dehydrates the mushrooms, while substantially preserving the mineral constituents, including ^137^Cs (Falandysz et al. 2019, 2020a, b; Falandysz et al. submitted for publication). Culinary processing of mushrooms, depending on the process, may or may not lead to a potential reduction of ^137^Cs and other contaminants in mushroom meals and is therefore a potentially important means of modulating human exposure.

This study aimed to study the effects of blanching and blanching followed by pickling on the leaching of ^137^Cs and ^40^K from processed fresh and frozen fruiting bodies of *Boletus edulis*. Additionally, dried mushrooms were also ground and soaked in cold water to assess possible leakage of the nuclides. Activity data obtained for the pickled *B. edulis* were also discussed relative to the activity concentrations of ^137^Cs and ^40^K in commercially pickled King Bolete and other mushrooms such as *Imleria badia* (Fr.) Fr. (Bay Bolete) and *Suillus luteus* (Slippery Jack).

## Materials and methods

### Materials

**Fig. 1:**
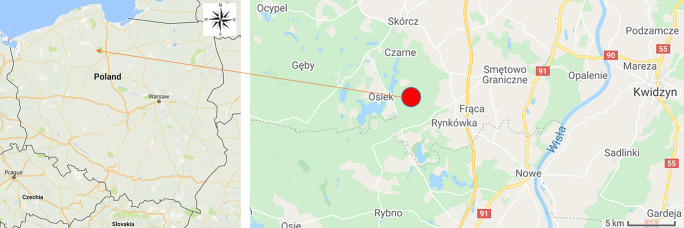
Localization of the sampling site of *B. edulis* nearby to the village of Osiek in the Tuchola Pinewoods from the north-central region of Poland (Google maps; colour figure available on-line)

Eighteen fruiting bodies of *Boletus edulis* (Bull: Fr.) were collected in the proximity of the village of Osiek in the Tuchola Pinewoods from the north-central region of Poland in 2015 (Fig. 1). The specimens were relatively large sized, well-developed, and in good body condition with white to yellow hymenophores. The mushrooms were carefully cleaned from foreign debris using a plastic knife and brush and separated into caps and stipes. The caps and stipes were cut using a plastic knife into three approximately equivalent pools. The separately pooled caps and stipes were subjected to household treatments including blanching; blanching and pickling; drying, grinding, and macerating; deep-freezing, blanching, and pickling.

In brief, batches of pooled fresh caps and stipes were subjected to blanching and blanching and pickling, and in parallel, a subsamples of pooled caps and stipes in sealed polyethylene bags were deep-frozen (− 20 °C) for a week. The frozen subsamples were subjected to blanching and blanching and pickling in the same manner as the fresh samples (Table 1). The procedures used for blanching (no salt) and pickling in diluted (proportion 1:4 of 100 mL) spirit vinegar (10%) were the same as described in a previous article (Drewnowska et al. 2017). The blanched and blanched and pickled mushrooms from each culinary experiment were lyophilised (model LYOVAC GT2; Steris, Germany) after draining the liquid, ground and stored in a dry condition until determination of ^137^Cs and ^40^K.

**Table 1:** ^137^Cs and ^40^K activity concentrations in dried King Bolete mushroom products and change in activity concentrations of nuclides after household processing (data based on a dry biomass)

Product and treatment	Caps	Stipes	Whole fruiting bodies	*Q*_C/S_	Caps	Stipes	Whole fruiting bodies
	^137^Cs (Bq kg^−1^ db)				Difference (%)		
Fresh → dried	370 ± 7	130 ± 4	270 ± 6	2.8	WD	WD	WD
Fresh → blanched	160 ± 4	65 ± 5	120 ± 4	2.5	− 56	− 50	− 55
Fresh → blanched → pickled	59 ± 3	24 ± 1	45 ± 2	2.5	− 84	− 81	− 83
Fresh → dried (powdered) → macerated	18 ± 2	7.2 ± 1	14 ± 1	2.5	− 95	− 94	− 95
Fresh → deep frozen → blanched	180 ± 3	44 ± 3	130 ± 3	4.1	− 51	− 66	− 52
Fresh → deep frozen → blanched → pickled	48 ± 2	< 9.3	~ 31	WD	− 87	− (~) 90	− (~)88
	^40^K (Bq kg^−1^ db)				Difference (%)		
Fresh → dried	750 ± 32	340 ± 50	590 ± 39	2.2	WD	WD	WD
Fresh → blanched	520 ± 39	200 ± 97	390 ± 62	2.6	− 40	− 41	− 34
Fresh → blanched → pickled	97 ± 36	46 ± 18	77 ± 29	2.1	− 87	− 86	− 87
Fresh → dried (powdered) → macerated	90 ± 50	43 ± 23	71 ± 39	2.1	− 88	− 87	− 88
Fresh → deep frozen → blanched	420 ± 31	190 ± 70	330 ± 47	2.2	− 44	− 44	− 44
Fresh → deep frozen → blanched → pickled	150 ± 40	68 ± 19	120 ± 32	2.2	− 80	− 80	− 80

*WD* without data

Unprocessed subsamples of caps and stipes were sliced using a plastic knife, then lyophilised, ground to a fine powder using a porcelain mortar and pestle and divided into two halves. One half served as a control to calculate the change in activity concentrations of ^137^Cs and ^40^K, and the second was used in a soaking (maceration) experiment. For this, aliquots of powdered caps and stipes (ca 1 g each) in a glass beaker (100 mL) were macerated for 24 h using cooled boiled water (50 mL) at room temperature. The macerate was separated from the fungal solids after filtration through a laboratory filter paper (medium fine) under gravity. The filter and solids were lyophilised, ground, and stored in sealed polyethylene bags in dry condition. Commercially pickled samples of King Bolete, Bay Bolete, and Slippery Jack in glass jars (250 mL) were purchased randomly from retail outlets in the city of Gdańsk in Poland in 2016. These commercially pickled mushrooms were drained from the marinade, lyophilized, ground, and stored in the same manner as the other studied materials, until analysis.

### Analysis

Directly before measurement of the ^137^Cs and ^40^K activities, the materials were deep-frozen and lyophilized for 3 days to remove any absorbed humidity. The activities were determined using a gamma spectrometer with a coaxial HPGe detector and with a relative efficiency of 18%. The resolution efficiency was 1.9 keV at 1.332 MeV (with associated electronics). The equipment was calibrated using a multi-isotope standard and the method was fully validated. The reference solution: “Standard solution of gamma emitting isotopes, code BW/Z-62/27/07” produced at the IBJ-Świerk near/Otwock in Poland, was used for preparing reference samples for equipment calibration. The same geometry of cylindrical dishes with 40 mm diameter (as applied for environmental samples) was used for reference samples during equipment calibration. Data obtained on activity concentration of ^137^Cs were decay corrected back to the time of sampling. The stable K contents were calculated from the ^40^K data (Falandysz et al. 2020a).

## Results and discussion

### Elemental composition

In order to track changes in the activity concentrations of ^137^Cs and ^40^K, and the total K concentration in processed *B. edulis*, and to estimate hypothetical intakes with a meal, an attempt has been made to express the results both on dry and whole (wet) weight basis (Tables 1 and 2 and Appendix). When assessing the risk from contaminants accumulated in mushrooms, but also the nutritional benefits, the expression of data both on a dry and whole weight is useful to better track the change in elemental content during the course of the household treatment. Thus, in realistic approaches, the intake and exposure assessments need to relate to the whole (wet) weight mushroom meal.

**Table 2:** ^137^Cs and ^40^K activity concentrations in King Bolete mushroom products and change in activity concentrations of nuclides after household processing [data presented on the whole (wet) weight basis]

Product and treatment	Caps	Stipes	Wholemushrooms	Caps	Stipes	Wholemushrooms
	^137^Cs (Bq kg^−1^ ww)			Difference (%)		
Fresh^#^	37 ± 1	13 ± 0	27 ± 1	WD	WD	WD
Fresh → blanched^*^	22 ± 1	9.0 ± 0.7	17 ± 1	− 40	− 31	− 37
Fresh → blanched → pickled	8.1 ± 0.4	3.3 ± 0.1	6.2 ± 0.3	− 78	− 31	− 77
Fresh → dried (powdered) → macerated	1.8 ± 0.2	0.72 ± 0.1	1.4 ± 0.2	− 95	− 94	− 95
Fresh → deep frozen → blanched	25 ± 1	1.9 ± 0.1	18 ± 1	− 32	− 85	− 67
Fresh → deep frozen → blanched → pickled	6.6 ± 0.3	< 1.3	~ 4.3	− 82	− (~)90	− (~)84
	^40^K (Bq kg^−1^ ww)			Difference		
Fresh	75 ± 3	34 ± 5	59 ± 4	WD	WD	WD
Fresh → blanched	72 ± 5	28 ± 14	54 ± 9	− 4.0	− 18	− 8.5
Fresh → blanched → pickled	13 ± 5	6.3 ± 2.5	11 ± 4	− 83	− 81	− 81
Fresh → dried (powdered) → macerated	9.0 ± 5.0	4.3 ± 2.3	7.1 ± 3.9	− 88	− 87	− 88
Fresh → deep frozen → blanched	58 ± 4	26 ± 10	46 ± 6	− 23	− 23	− 22
Fresh → deep frozen → blanched → pickled	21 ± 6	9.4 ± 2.6	17 ± 5	− 72	− 72	− 72

*WD* without data

^#^Moisture 90.0%; ^*^moisture 90.44% (Jaworska and Bernaś 2009)

*B. edulis* from the Tuchola Pinewoods in 2015 showed relatively low contamination with ^137^Cs in the whole fruiting bodies at a level of 270 Bq kg^−1^ db (27 Bq kg^−1^ ww), while higher activity was noted for the natural radionuclide ^40^K at 590 Bq kg^−1^ db (59 Bq kg^−1^ ww) (Tables 1 and 2). The mushrooms’ caps usually are more contaminated with ^137^Cs than stipes (Falandysz and Borovička 2013; Falandysz et al. 2018). In this study, the *B. edulis* was characterised by a ^137^Cs activity quotient (*Q*_C/S_—of activity concentration in caps compared with stipes) of 2.8, with 2.1 to 4.5 for the cooked products on a dry biomass basis. Corresponding values for ^40^K were 2.2 (2.1 to 4.1 in cooked products) (Table 1). In *B. edulis* the caps usually show higher concentrations of K than the stipes and the reported median *Q*_C/S_ from another study was 1.5 (Frankowska et al. 2010). Radionuclide ^40^K (*t*_1/2_ is 1.248 × 10^9^ years) is a minor part of the natural K that is the major metallic element in mushrooms (atomic percent occurrence is 0.0117%) (Falandysz and Borovička 2013; Falandysz et al. 2020a; Frankowska et al. 2010). Estimated total K concentration in the whole fruiting bodies of *B. edulis* in this study was 21,000 mg kg^−1^ db and 2100 mg kg^−1^ ww. Drying of *B. edulis* results in an up to tenfold increase in the content of minerals relative to the original product.

In this study fresh *B. edulis* when blanched showed a ^137^Cs activity concentration of 120 Bq kg^−1^ db (a decrease of 55% relative to the fresh product) equivalent to17 Bq kg^−1^ ww (decrease of 37%). ^40^K activity was recorded at 390 Bq kg^−1^ db (decrease of 34%) and 54 Bq kg^−1^ ww (decrease of 8.5%) (Tables 1 and 2). The fresh mushrooms when blanched and pickled showed ^137^Cs activity concentration of 45 Bq kg^−1^ db (a decrease of 83%) and 6.2 Bq kg^−1^ ww (decrease of 77%). ^40^K activity was 77 Bq kg^−1^ db (a decrease of 87%) and 11 Bq kg^−1^ ww (decrease of 81%) (Tables 1 and 2). The total K contents in fresh *B. edulis* when blanched were 14,000 mg kg^−1^ db (1900 Bq kg^−1^ ww), and when blanched and pickled, were 2700 mg kg^−1^ db (370 mg kg^−1^ ww) (Appendix).

When fresh *B. edulis* was deep frozen for a period of one week (− 20 °C) and then blanched, the ^137^Cs at activity concentration was 130 Bq kg^−1^ db (18 Bq kg^−1^ ww) and the^40^K activity concentrations was 330 Bq kg^−1^ db (46 Bq kg^−1^ ww). The total K content was 8800 mg kg^−1^ db and 1200 mg kg^−1^ ww (Tables 1 and 2 and Appendix). Fresh *B. edulis* when deep frozen and next blanched and pickled had ^137^Cs at activity concentration of 31 Bq kg^−1^ db (4.3 Bq kg^−1^ ww), and the activity of ^40^K was 120 Bq kg^−1^ db (17 Bq kg^−1^ ww) (Tables 1 and 2). The total K content was 3200 mg kg^−1^ db (440 mg kg^−1^ ww) (Appendix). The ^137^Cs and ^40^K leaching rates from deep frozen and blanched or blanched and pickled mushrooms were 88 and 80% (db), equivalent to 84 and 72% (ww) respectively (Tables 1 and 2).

As mentioned, blanching decreases activities of ^137^Cs and ^40^K and contents of total K in processed mushrooms in relation to raw substrate mushrooms when the data are expressed on a dry weight (biomass) basis (Table 1). In this study the degree of reduction and hypothetical intake of the elements differed depending on whether the results were estimated on a dry biomass basis or on a whole (wet) weight. Blanched, fresh mushrooms lost ^137^Cs at 55% db and depending on the morphological part, at 4 to 37% on a ww basis, while deep frozen and blanched mushrooms lost 52% db (67% ww). The loss of potassium during blanching of fresh mushrooms was 34% in db, and if expressed on a whole weight, the loss was 8.5%. When deep frozen and blanched, the reduction of K was 44% (db) and 22% (ww) (Table 2 and Appendix).

Mushrooms collected from the wild may represent an insignificant component of the diet, but they can make a significant contribution to the intake of radiocaesium (Kiefer et al. 1965; Rantavaara 2003). Hence, cooking procedures that decrease the content of radiocaesium in mushroom are beneficial. Blanching is necessary in some cooking recipes of mushrooms. The household blanching of mushrooms retains a portion of ^137^Cs within the blanched product (Tables 1 and 2) and results obtained in this study are in good agreement with the findings by Daillant et al. (2013). Frying decreases the moisture content and causes retention of a portion of ^137^Cs in the cooked mushrooms, and in consequence, the fried product relative to the fresh material on a whole (wet) weight basis, can be enriched in this nuclide (Falandysz et al. 2020a, b). There is no previous data available on the reduction of potassium in mushrooms through the effects of blanching or blanching and pickling. Activity concentration of ^137^Cs in macerated mushrooms was at 14 Bq kg^−1^ db (1.4 Bq kg^−1^ ww), and the reduction in content when related to the fresh substrate was 95% (assuming 90% moisture in rehydrated product). It appears that the degree of disintegration of fruiting bodies is a major factor determining the rate at which mineral constituents leach from cells. Traditionally, the soaking period varies from 2 h to overnight (12 h) regardless of whether the powdered or sliced product is used. In practice, the rehydration yield could be < 90% if the whole dried fruiting bodies were rehydrated, due to an effect of maximal shrinkage as well as a decrease in rehydration ability in this state, while for sliced or powdered states, the rehydration yield can be higher.

For comparison with other species, the fresh fruiting bodies of *Cantharellus tubaeformis* soaked for 12 h (200 g in 3 L fresh water) lost 40% radioactivity from ^137^Cs, increasing to 50% and 61% in salted water (1% and 5% NaCl respectively) (the organoleptic values were not affected very much). The loss increased to 95% after twice rinsing and blanching, but this caused the resulting product to have a slimy consistency (Stijve 1994). Whole, dried *C. tubaeformis* (16 g) macerated for 30 min (0.5 L water) lost 40% of ^137^Cs. However, when macerated for 15 min (0.5 L water) and then blanched for 3 min, the sample lost 99% of ^137^Cs (but this procedure preserved good texture and taste in the reconstituted mushrooms) (Stijve 1994). Dried *Craterellus lutescens* (Fr.) Fr. (previous name *Cantharellus lutescens* Fr.) and *Cortinarius caperatus* (Pers.) Fr., (previous name *Rozites caperatus* (Pers.) P. Karst.) soaked in water for three hours lost 70% of ^137^ Cs on a dry weight biomass basis (Daillant et al. 2013). Soaking of dried shitake mushrooms in water decreased the radiocesium content by around 50% when compared with uncooked shitake (Nabeshi et al. 2013).

In this study, mushrooms after maceration, contained an activity level of ^40^K at 71 Bq kg^−1^ db (7.1 Bq kg^−1^ ww) with a total K content of 2500 mg kg^−1^ db (250 mg kg^−1^ db). The rate of decrease was 88%. Results from this study confirm some findings by Daillant et al. (2013), that removal of ^137^Cs using blanching and fresh boiling water is only partially successful, while on the other hand, it retains some of the potassium. Both ^137^Cs and ^40^K decreased more or less at a similar rate under the household processes used in this study. Decrease of ^137^Cs and ^40^K was roughly lower if data were calculated based on the whole (wet) weight of cooked mushrooms than on dried biomass.

Activities of ^137^Cs in cooked mushrooms (potential meals) from 2015 were in the range of 1.4 to 25 Bq kg^−1^ ww, which is considerably lower than the maximum permitted value of 600 Bq kg^−1^ that is considered as a safe concentration in foodstuffs (Falandysz et al. 2020a). When considered in parallel, the activities from ^40^K were in the range of 7.1 ± 3.9 to 72 ± 5 Bq kg^−1^ ww, i.e., 3 to 5-fold higher (Table 2). The estimated potassium content in processed *B. edulis* varied from 250 ± 140 to 1900 ± 320 mg kg^−1^ ww, depending on the processing mode (Appendix).

If it is assumed that a 100-g whole (wet) weight portion of blanched or pickled mushrooms represents a single mushroom meal, it can provide from 25 to 460 (median 120) mg of K. This mushroom dish containing 120 mg K accounts for around 3% of the adequate daily adult intake, assuming absorption at around 90% (recommended intake is 4700 mg) (NIH 2020). Fried mushrooms on average seem to be a much better source of K intake than mushrooms that are blanched and then pickled (Falandysz et al. 2020a, b). In this study, fresh mushrooms when blanched alone could provide from 120 to 460 (median 190) mg of K in a 100-g meal. Commercially pickled *B. edulis*, *I. badia*, and *S. luteus* showed ^137^Cs at activity concentrations of 10 ± 1.0 Bq kg^−1^ db, 34 ± 1.0 Bq kg^−1^ db and < 0.53 Bq kg^−1^ db, respectively. On a whole weight, the ^137^Cs activity concentrations in commercially pickled products was 1.5 ± 0.1 Bq kg^−1^ ww in *B. edulis*, 4.6 ± 0.1 Bq kg^−1^ ww in *I. badia*, and < 0.07 Bq kg^−1^ ww in *S. luteus*. The moisture content of these products was in the range of 85.4 to 86.7%. Thus, the contamination levels in commercial products were much lower than those seen for *B. edulis* in this study. Analogically to ^137^Cs, the activity concentrations from ^40^K and the contents of total K in the commercial products were 50 ± 10 Bq kg^−1^ db and 7.3 ± 1.5 Bq kg^−1^ ww and 1600 ± 300 mg kg^−1^ db and 230 ± 50 mg kg^−1^ ww (*B. edulis*), 95 ± 11 Bq kg^−1^ db and 13 ± 2 Bq kg^−1^ ww and 3100 ± 400 mg kg^−1^ db and 410 ± 60 mg kg^−1^ ww (*I. badia*), and 81 ± 11 Bq kg^−1^ db and 11 ± 1 Bq kg^−1^ ww and 2600 ± 400 mg kg^−1^ db and 350 ± 30 mg kg^−1^ ww (*S. luteus*).

## Conclusions

This study confirms earlier reports that blanching of fresh mushrooms using traditional methods of household preparation is to some degree efficient at removing ^137^Cs. The initial rate of fruiting body disintegration and pre-preparation can have an impact on the leaching rate of the water-soluble fraction of metallic elements. Mushrooms that are uncontaminated or that show low concentrations of radiocaesium can still retain some potassium when subjected to blanching and pickling. Blanching of the fungal materials always decreased activities resulting from ^137^Cs and ^40^K, but also the total K content of the product, relative to the substrate, if data were expressed on a dry weight (biomass) basis.

## Electronic supplementary material

ESM 1 (DOCX 17 kb)

## Data Availability

Not applicable
