# Supplementary material for: The effects of different cooking modes on the 137Cs, 40K, and total K content in Boletus edulis (King Bolete) mushrooms
Source: Environ Sci Pollut Res Int. 2020 Oct 19;28(10):12441–6. doi: 10.1007/s11356-020-11147-7 (PMC7921016; doi:10.1007/s11356-020-11147-7)
Supplement: Supplementary file 1 — (DOCX 17 kb) [file 11356_2020_11147_MOESM1_ESM.docx]

Appendix 1. Estimated K concentrations in King Bolete before and after culinary processing

| Product and treatment | K (mg kg^-1^ db) | | | K (mg kg^-1^ ww) | | |
| --- | --- | --- | --- | --- | --- | --- |
|  | Caps | Stipes | Whole mushrooms | Caps | Stipes | Whole mushrooms |
| Fresh → dried^#^ | 27000 ± 1100 | 12000 ± 1800 | 21000 ± 1400 | 2700 ± 110 | 1200 ± 180 | 2100 ± 140 |
| Fresh → blanched | 19000 ± 1400 | 7100 ± 3400 | 14000 ± 2200 | 2600 ± 180 | 980 ± 490 | 1900 ± 320 |
| Fresh → blanched → pickled | 3500 ± 1300 | 1600 ± 630 | 2700 ± 1000 | 480 ± 180 | 220 ± 87 | 370 ± 130 |
| Fresh → dried (grounded) → macerated | 3200 ± 1800 | 1500 ± 800 | 2500 ± 1400 | 320 ± 180 | 150 ± 80 | 250 ± 140 |
| Fresh → deep frozen → blanched | 15000 ± 1100 | 6800 ± 2500 | 8800 ± 1200 | 2100 ± 140 | 940 ± 360 | 1200 ± 160 |
| Fresh → deep frozen → blanched → pickled | 5400 ± 1400 | 2400 ± 670 | 3200 ± 850 | 740 ± 210 | 330 ± 91 | 440 ± 130 |

Notes: ^#^Moisture content of fresh mushrooms in good physical condition is 90% (consensus value), hence, content of the mineral constituents in dehydrated mushrooms is around tenfold greater than in fresh specimens.
